# Correlation of androgen receptor with ultrasound, clinicopathological features and clinical outcomes in breast cancer

**DOI:** 10.1186/s13244-023-01387-9

**Published:** 2023-03-16

**Authors:** Xudong Zhang, Hao Cui, Nana Hu, Peng Han, Wei Fan, Panting Wang, Xiaoxuan Zuo, Dantong Zhao, He Huang, Shuo Li, Hanqing Kong, Fuhui Peng, Jiawei Tian, Lei Zhang

**Affiliations:** 1 Department of Ultrasound Medicine, The Second Affiliated Hospital of Harbin Medical University, Harbin, Heilongjiang China 150086; 2 Department of Clinical Medicine, The Second Affiliated Hospital of Harbin Medical University, Harbin, Heilongjiang China 150086

**Keywords:** Breast cancer, Androgen receptor, Ultrasound, Nomogram, Prognosis

## Abstract

**Background:**

This study aimed to explore whether there is an association between androgen receptor (AR) expression and ultrasound, clinicopathological features and prognosis of breast cancer.

**Methods:**

A total of 141 breast cancer patients were included in this retrospective study. AR expression was analyzed by immunohistochemistry. The images of B-mode, color Doppler and strain elastography from 104 patients were collected continuously, and the corresponding ultrasound characteristics were obtained. The differences in ultrasound and clinicopathological features in different AR status were analyzed. Progression-free survival (PFS) of patients was obtained through up to 90 months of follow-up; then, the effect of AR on PFS was analyzed. Subsequently, a nomogram was constructed to predict the AR status. The predictive accuracy was calculated using C-index.

**Results:**

The positive expression of AR (AR +) was associated with lower histological grade (*p* = 0.034) and lower Ki-67 level (*p* = 0.029). Triple-negative breast cancer (TNBC) had the lowest probability of AR + (*p* < 0.001). The AR + group mostly showed unsmooth margin (*p* < 0.001), posterior acoustic shadowing (*p* = 0.002) and higher elasticity score (*p* = 0.022) on ultrasound. The echo pattern of most tumors with AR + was heterogeneous (*p* = 0.024) in Luminal A subtype. AR + could be a sign of a better prognosis in overall breast cancer (*p* < 0.001), as well as in human epidermal growth factor receptor 2 (HER2) overexpression and Luminal B subtypes (*p* = 0.001 and 0.025). The nomogram showed relatively reliable performance with a C-index of 0.799.

**Conclusion:**

Our research demonstrated that AR expression was closely related to ultrasound, clinicopathological features and prognosis of breast cancer.

**Supplementary Information:**

The online version contains supplementary material available at 10.1186/s13244-023-01387-9.

## Key points

- In terms of clinicopathology, the probability of AR expression in different subtypes of breast cancer was various, and the positive expression of AR was associated with lower histological grade and lower Ki-67 level.
- In terms of ultrasound, the positive expression of AR was associated with unsmooth margin, posterior acoustic shadowing and stiffer on strain elastography, and AR + and AR– tumors performed differently when analyzed separately according to different molecular subtypes.
- The positive expression of AR could be a sign of a better prognosis in breast cancer.

**Supplementary Information:**

The online version contains supplementary material available at 10.1186/s13244-023-01387-9.

## Introduction

Breast cancer, with a global incidence of approximately 2.26 million new cases each year, has overtaken lung cancer to become the most commonly diagnosed cancer globally, which seriously threatens women's health and life [1]. Meanwhile, a proportion of patients still cannot benefit from current clinical treatments due to drug resistance, lack of effective treatment targets and other factors [2, 3]. This makes the diagnosis and treatment of breast cancer increasingly challenging. Consequently, it is urgent to explore new molecular markers and potential therapeutic targets for breast cancer to improve the prognosis. Breast cancer is a highly hormone dependent tumor [4]. As a member of the steroid receptor in the nuclear receptor superfamily, the androgen receptor (AR) plays a vital role in breast cancer, together with the estrogen receptor (ER) and progesterone receptor (PR) [5]. Moreover, it is widely expressed in osteosarcoma, tissues of the prostate, liver, cardiovascular, breast and other human tissues, among which the expression of AR in breast cancer is the third highest [6]. Recently, the development of selective androgen receptor modulators, the great effect of AR–related therapy in prostate cancer and the in-depth study of luminal androgen receptor (LAR) subtype of breast cancer have highlighted AR. Several studies have confirmed that AR can be used as a potential therapeutic target and an emerging prognostic marker to guide the clinical treatment of breast cancer [7].

The expression of molecular markers affects the biological and histological behavior of breast cancer and then affects the imaging appearances; they are inextricably related [8, 9]. Ultrasound and mammography are two distinct imaging methods. They are complementary to each other and both play the important role in the diagnosis and treatment of breast diseases [10]. Ultrasound has a variety of modalities, B-mode can show the shape of the mass, internal echo and other two-dimensional features, color Doppler can reflect the blood perfusion of the tumors, and elastography can assess the hardness of the tumors, so that, ultrasound can identify benign and malignant tumors, predict axillary lymph node metastasis and guide percutaneous biopsy and interventional therapy, among others [11, 12]. Current research focuses on the relationship between ultrasound characteristics and molecular biological expression [13]. Liu et al. [14] found that the positive expression of human epidermal growth factor receptor 2 (HER2) was related to the blood supply, lymph node metastasis and microcalcification. ER + was correlated with tumor morphology, margin and perimeter. Similarly, Zhao et al. reported that the positive expression of Ki-67 was associated with tumor diameter, blood flow grade and lymph node metastasis [15]. However, few studies have explored the relationship between ultrasound appearances and the expression of AR.

Bae et al. [16] suggested that AR + was related to calcifications with or without a mass on mammography, non-mass enhancement on MRI, irregular shape or spiculated margins on ultrasound. Candelaria et al. [17] found that the majority of the mammography of TNBC with AR + showed heterogeneously dense breast composition and high mass density, and the ultrasound showed irregular mass shape. Muller et al. [18] reported that most LAR tumors showed spiculated margins on mammography and smooth borders on ultrasound. Such studies related to AR have primarily focused on TNBC, instead of other molecular subtypes. Therefore, in this retrospective research, we aimed to determine whether AR status was related to the ultrasound, clinicopathological features or prognosis of breast cancer and to demonstrate whether such a correlation existed in different molecular subtypes.

## Materials and methods

### Patients

**Fig. 1:**
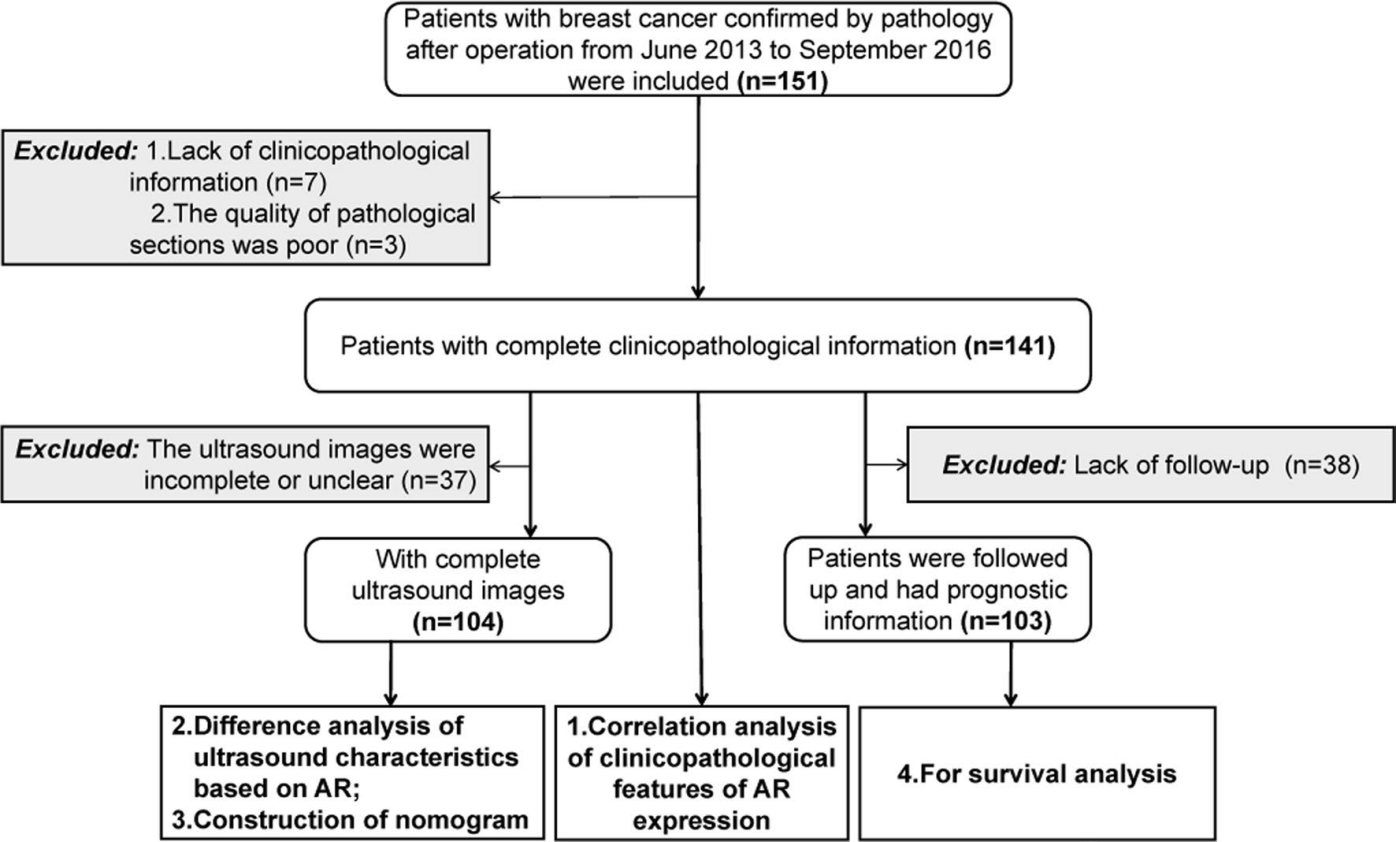
Flow chart for patient selection. *AR*, androgen receptor

This is a retrospective study of case information collated from June 2013 to September 2016. A total of 151 breast cancer patients underwent ultrasound examination before the operation and obtained pathological sections postoperatively. However, 7 of these patients lacked clinicopathological information and the other 3 patients had poor quality of pathological sections, which led to the exclusion of these 10 patients. Finally, 141 patients were included in the study. This study was approved by the institutional ethics committee of Harbin medical university (approval number, KY-2016-127). We reviewed the clinical data and the ultrasound images of these patients during the study period. The process of selecting patients for data analysis is presented in Fig. 1.

### Baseline data collection

We reviewed the following data from the 141 patients’ medical records: age, tumor size, menopausal status (non-menopause and menopause), lymph node metastasis (absent and present), distant metastasis (absent and present), histological type (invasive ductal carcinoma and other types), histological grade (1, 2 and 3) [19] and clinical TNM stage (cT1, cT2, cT3 and cT4) [20]. According to the immunohistochemistry (IHC) results for ER, PR, Ki-67 and HER2 expression, breast cancer was divided into the following four molecular subtypes: Luminal A (ER + and/or PR + , HER2-, Ki-67 < 14%); Luminal B (ER + and/or PR + , HER2-, Ki-67 ≥ 14%; ER + and/or PR + , HER2 overexpressed); HER2 overexpression (ER-, PR-, HER2 overexpressed); TNBC (ER-, PR-, HER2-) [21].

### Ultrasound examination

The ultrasound images of breast masses in 104 patients were scanned by a radiologist with 5 years of experience in breast ultrasound using a HITACHI Vision 500 system (Hitachi Medical System, Tokyo, Japan) equipped with a linear probe of 5–13 MHz. In real-time scanning, the static images of the maximum diameter of the mass during transverse and longitudinal cutting, and the dynamic images of the standard sections of B-mode, color Doppler and ultrasound strain elastography were preserved. These ultrasound images were reviewed independently by two breast radiologists with the wealth of experience of 8 and 10 years, respectively, while ignoring the IHC results. Where differences occurred, a consensus was reached. Feature extraction of B-mode ultrasound images was based on the Breast Imaging Reporting and Data System (BI-RADS) lexicon [22]. The ultrasound characteristics were included as follows: shape, orientation, hyperechoic halo, margin, posterior acoustic pattern, calcification, echo pattern, adler grade and elasticity score. 5-point elasticity scoring, as one of the evaluation systems, is highly specific in evaluating the stiffness of breast lesions [23]. The evaluation of ultrasound elastography was based on the World Federation of Ultrasound in Medicine and Biology (WFUMB) [24]. Blood flow was assessed according to Adler grade (0, 1, 2 and 3) [25].

### Immunohistochemistry

The expression of AR was analyzed by IHC based on tissue microarray (TMA), which was evaluated by two pathologists with > 10 years of breast pathology experience. The TMA section was stained with AR antibody (clone AR 441, DAKO). Normal breast tissue on TMA was used for internal control. We choose known positive breast cancer tissues as positive controls. Negative controls were prepared by omitting the primary antibody. False negatives and false positives were avoided by this approach, similar to ER and PR. AR positivity is defined as ≥ 10% of tumor cells with positive nuclear staining [26]. According to the American Society of Clinical Oncology/College of American Pathologists (ASCO/CAP) guideline, the cutoff value of ER and PR positivity was defined as 1% [27]. The IHC score of HER2 status included 0, 1 + , 2 +  and 3 +  and HER2 overexpression was defined by 3 + or 2 + with a > twofold change in fluorescence in situ hybridization (FISH), while 0 and 1 + were defined as HER2 negativity [28]. Positive nuclear staining of Ki-67 ≥ 14% was defined as high expression and < 14% was defined as low expression [29].

### Patient follow-up

Of the 141 patients, 103 were followed up in multiple methods. We gave priority to collecting as much detailed as possible through outpatient review information, inpatient treatment records, followed by telephone follow-ups. These patients were followed up twice a year with a 6-month cycle. Patients without recurrence were followed for at least 60 months, and those with recurrence were followed until the time of recurrence. Progression-free survival (PFS) was used as the study endpoint and was defined as the time from the operation date to tumor recurrence and death or the last follow-up date.

### Statistical analysis

IBM SPSS Statistics, Version 26.0, and R Version 4.1.1 (http://www.R-project.org) were used for statistical analysis. The relationship between clinicopathological features and AR status was analyzed by univariate and multivariate logistic regression analysis; the odds ratio (OR) and 95% confidence interval (95% CI) were calculated, and the differences in ultrasound characteristics between AR + and AR– groups were evaluated using the Chi-square test or Fisher's exact test. A nomogram based on the logistic regression analysis model was constructed to predict AR status. The efficiency of the logistic regression model was calculated by receiver operating characteristic curve (ROC) and area under curve (AUC). Additionally, the performance of the nomogram was measured by C-index and calibration curve. Moreover, the Kaplan–Meier method was used to evaluate PFS. Multivariate Cox proportional hazards model was used to analyze prognostic factors. For all the analysis *p* values < 0.05 were considered statistically significant.

## Results

### The correlation between the expression of AR and clinicopathological features

**Fig. 2:**
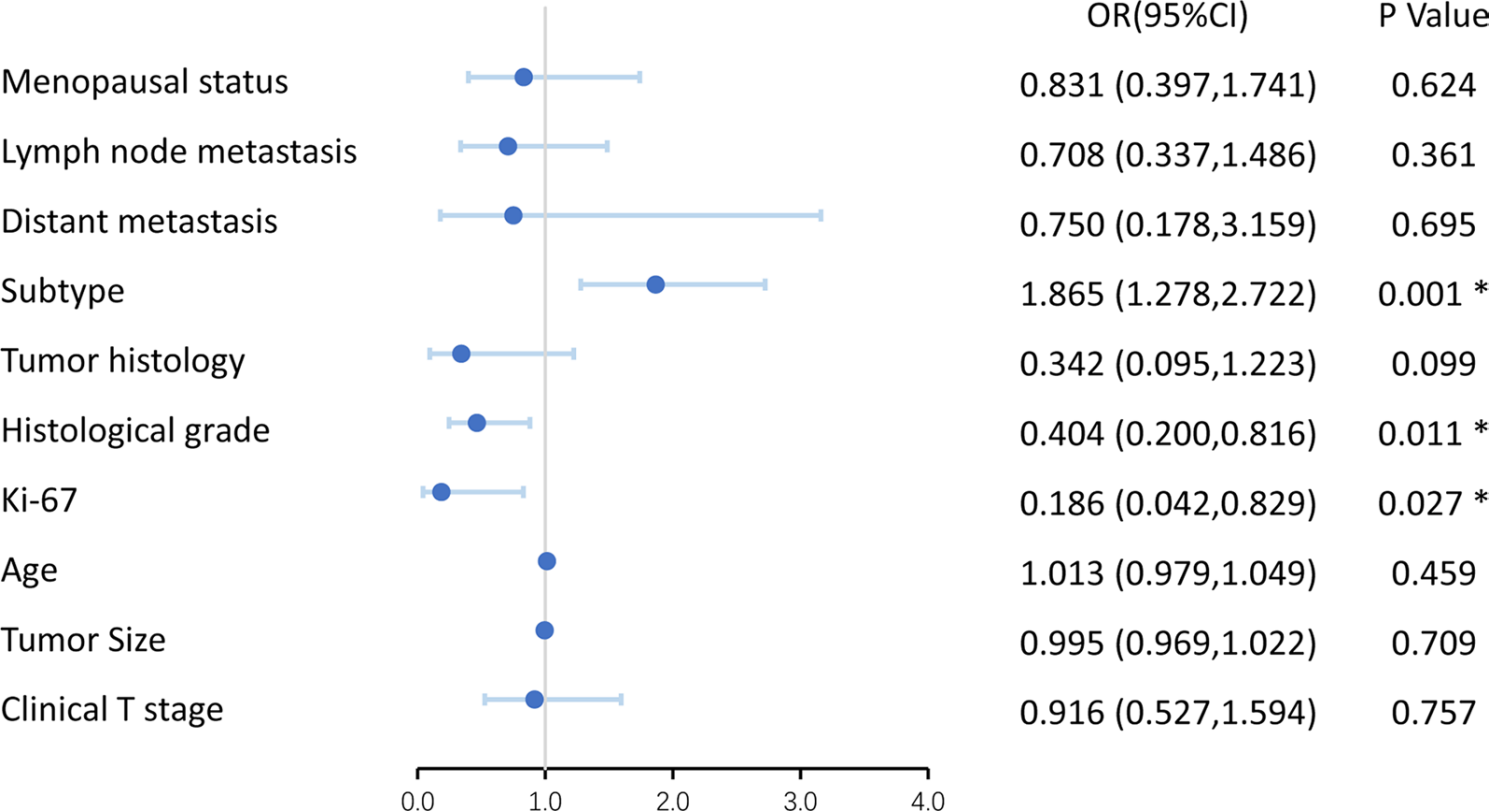
Forest map of univariate logistic regression analysis based on clinicopathological features related to AR. *OR*, odds ratio; *CI*, confidence interval; *AR*, androgen receptor; * indicates *p* < 0.05

As presented in Table 1, of the 141 breast cancer patients in our study, 102 (72.34%) were AR + and 39 (27.66%) were AR–. The mean age of AR + was 52.25 ± 11.29 years (range, 34–83), and that of AR– was 50.72 ± 10.12 years (range, 33–73) at the time of diagnosis. The average size of tumor in AR + was 22.98 ± 14.46 mm (range, 7–70) and in AR– was 23.95 ± 12.09 mm (range, 7–51). A total of 55 tumors could be palpated obviously, all larger than 2 cm. We found that molecular subtype (OR: 1.865 (1.278, 2.722), *p* = 0.001), histological grade (OR: 0.404 (0.200, 0.816), *p* = 0.011) and Ki-67 level (OR: 0.186 (0.042, 0.829), *p* = 0.027) were significantly different between the two groups by univariate logistic regression analysis (Fig. 2). In our study, TNBC had the lowest AR positive expression probability (18.18%, 4/22, *p* < 0.001); further, AR + was related to lower histological grade (OR: 0.459 (0.223, 0.943), *p* = 0.034) and lower Ki-67 level (OR: 0.177 (0.037, 0.839), *p* = 0.029) by multivariate logistic regression analysis.

**Table 1:** Summary data of clinicopathological features of 141 patients

Characteristics	AR + population (*n* = 102)		AR– population (*n* = 39)		*p* value
	*n*	Percent (%)	*n*	Percent (%)	
Age (y)					0.459
Mean ± SD	52.25 ± 11.29		50.72 ± 10.12		
Subtype					0.001*
TNBC	4	3.92	18	46.15	
Luminal A	36	35.29	6	15.38	
Luminal B	31	30.39	4	10.26	
HER2 overexpression	31	30.39	11	28.21	
Ki-67 (%)					0.027*
< 14	23	22.55	2	5.13	
≥ 14	79	77.45	37	94.87	
Tumor size (mm)					0.709
Mean ± SD	22.98 ± 14.46		23.95 ± 12.09		
Lymph node metastasis					0.361
Absent	61	59.80	19	48.72	
Present	41	40.20	20	51.28	
Menopausal status					0.624
Non-menopause	45	44.12	19	48.72	
Menopause	57	55.88	20	51.28	
Distant metastasis					0.695
Absent	96	94.12	36	92.31	
Present	6	5.88	3	7.69	
Tumor histology					0.099
Invasive ductal carcinoma	82	80.39	36	92.31	
Other types	20	19.61	3	7.69	
Clinical T stage					0.757
cT1	62	60.78	20	51.28	
cT2	29	28.43	17	43.59	
cT3 and cT4	11	10.78	2	5.13	
Histological grade					0.011*
1	12	11.76	1	2.56	
2	50	49.02	15	38.46	
3	40	39.22	23	58.97	

*y*, years old; *TNBC*, triple-negative breast cancer; *HER2*, human epidermal growth factor receptor 2; *AR*, androgen receptor; * indicates *p* < 0.05

### The comparison of the ultrasound characteristics of the groups with different AR status

**Fig. 3:**
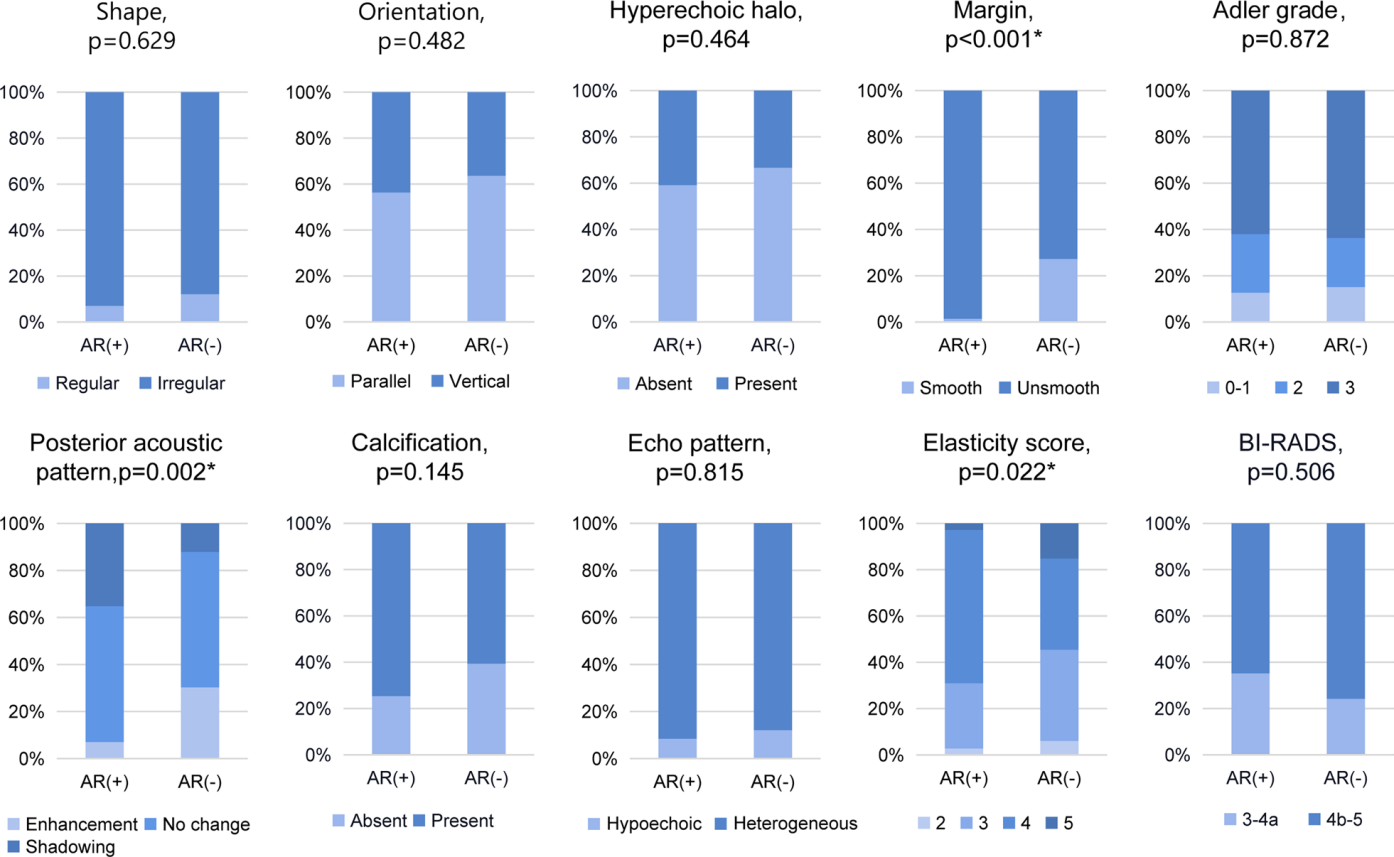
Comparison of ultrasound characteristics between AR + and AR– groups in the entire study cohort. *BI-RADS*, Breast Imaging Reporting and Data System; *AR*, androgen receptor; * indicates *p* < 0.05

**Fig. 4:**
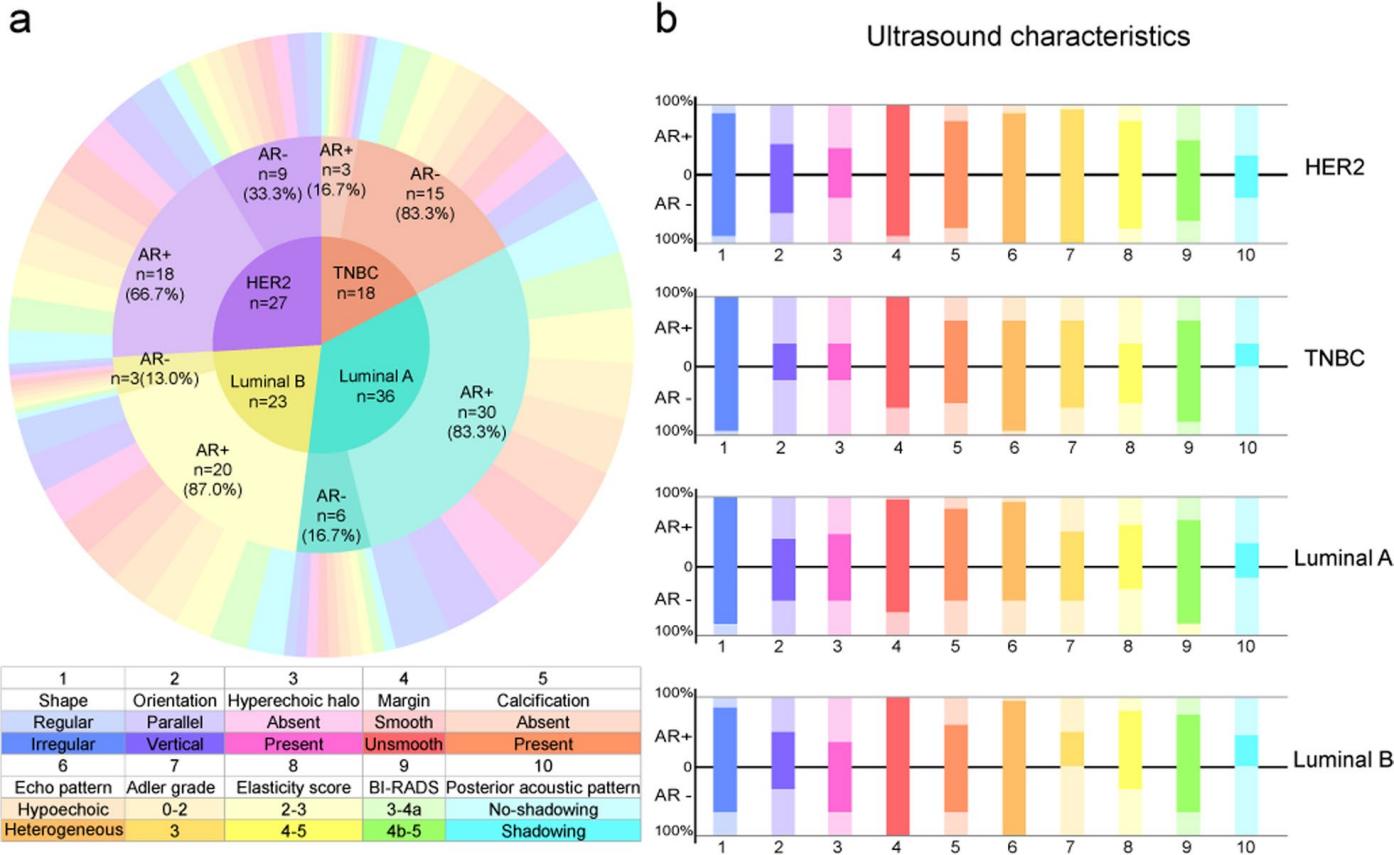
Comparison of ultrasound characteristics between AR– and AR + groups in different molecular subtypes. **a** Expression of AR in different molecular subtypes. **b** The distribution differences of ultrasound characteristics between AR– and AR + groups in HER2 overexpression, TNBC, Luminal A and Luminal B subtypes, respectively. *TNBC*, triple negative breast cancer; *HER2*, human epidermal growth factor receptor 2; *AR*, androgen receptor; *BI-RADS*, Breast Imaging Reporting and Data System

The ultrasound characteristics of AR + and AR– breast cancer were significantly different. On ultrasound, 98.59% (70/71) of AR + breast cancer showed unsmooth margin on ultrasound; however, that of AR– was 72.73% (24/33). Only 1 of 71 cases of AR + breast cancer showed smooth margin (*p* < 0.001). Compared with AR– breast cancer, AR + was more likely to be posterior acoustic shadowing (35.21% vs 12.12%, *p* = 0.002). The elasticity score of AR + breast cancer was mostly concentrated in 4 (66.20% vs 39.39%, *p* = 0.022) (Fig. 3, Table 2 and Additional file 1: Fig. S1). The analysis of the relationship between AR and ultrasound characteristics in different molecular subtypes showed that: in the Luminal A subtype, with the different expression of AR, the echo pattern of the tumor was different (*p* = 0.024). The internal echo pattern in AR + group was more heterogeneous. However, in our study, perhaps because the sample size was not large enough, no significant differences were found in ultrasound characteristics when AR expression differed in the other three subtypes (Fig. 4 and Table 3).

**Table 2:** Ultrasound characteristics of AR status in 104 patients

Characteristics	AR + (*n* = 71)	AR– (*n* = 33)	*χ*^2^value	*p* value
Shape			0.233	0.629
Regular	5 (7.04%)	4 (12.12%)		
Irregular	66 (92.96%)	29 (87.88%)		
Orientation			0.495	0.482
Parallel	40 (56.34%)	21 (63.64%)		
Vertical	31 (43.66%)	12 (36.36%)		
Hyperechoic halo			0.537	0.464
Absent	42 (59.15%)	22 (66.67%)		
Present	29 (40.85%)	11 (33.33%)		
Margin			14.493	< 0.001*
Smooth	1 (1.41%)	9 (27.27%)		
Unsmooth	70 (98.59%)	24 (72.73%)		
Posterior acoustic pattern			12.759	0.002*
Enhancement	5 (7.04%)	10 (30.30%)		
No change	41 (57.75%)	19 (57.58%)		
Shadowing	25 (35.21%)	4 (12.12%)		
Calcification			2.123	0.145
Absent	18 (25.35%)	13 (39.39%)		
Present	53 (74.65%)	20 (60.61%)		
Echo pattern			0.055	0.815
Hypoechoic	6 (8.45%)	4 (12.12%)		
Heterogeneous	65 (91.55%)	29 (87.88%)		
Adler grade			0.273	0.872
0–1	9 (12.68%)	5 (15.15%)		
2	18 (25.35%)	7 (21.21%)		
3	44 (61.97%)	21 (63.64%)		
Elasticity score			9.429	0.022*
2	2 (2.82%)	2 (6.06%)		
3	20 (28.17%)	13 (39.39%)		
4	47 (66.20%)	13 (39.39%)		
5	2 (2.82%)	5 (15.15%)		
BI-RADS			0.443	0.506
3-4a	25 (35.21%)	8 (24.24%)		
4b-5	46 (64.79%)	25 (75.76%)		

*BI-RADS*, Breast imaging-reporting and data system; *AR*, androgen receptor; * indicates *p* < 0.05

**Table 3:** Ultrasound characteristics of AR status in different molecular subtypes

Characteristics	TNBC (*n* = 18)			Luminal A (*n* = 36)			Luminal B (*n* = 23)			HER2 overexpression (*n* = 27)		
	AR + (*n* = 3)	AR– (*n* = 15)	*p* value	AR + (*n* = 30)	AR– (*n* = 6)	*p* value	AR + (*n* = 20)	AR– (*n* = 3)	*p* value	AR + (*n* = 18)	AR– (*n* = 9)	*p* value
Shape			1.000			0.167			0.453			1.000
Regular	0 (0.0%)	1 (6.7%)		0 (0.0%)	1 (16.7%)		3 (15.0%)	1 (33.3%)		2 (11.1%)	1 (11.1%)	
Irregular	3 (100.0%)	14 (93.3%)		30 (100.0%)	5 (83.3%)		17 (85.0%)	2 (66.7%)		16 (88.9%)	8 (88.9%)	
Orientation			1.000			0.677			1.000			0.695
Parallel	2 (66.7%)	12 (80.0%)		18 (60.0%)	3 (50.0%)		10 (50.0%)	2 (66.7%)		10 (55.6%)	4 (44.4%)	
Vertical	1 (33.3%)	3 (20.0%)		12 (40.0%)	3 (50.0%)		10 (50.0%)	1 (33.3%)		8 (44.4%)	5 (55.6%)	
Hyperechoic halo			1.000			1.000			0.538			1.000
Absent	2 (66.7%)	12 (80.0%)		16 (53.3%)	3 (50.0%)		13 (65.0%)	1 (33.3%)		11 (61.1%)	6 (66.7%)	
Present	1 (33.3%)	3 (20.0%)		14 (46.7%)	3 (50.0%)		7 (35.0%)	2 (66.7%)		7 (38.9%)	3 (33.3%)	
Margin			0.515			0.066			/			0.333
Smooth	0 (0.0%)	6 (40.0%)		1 (3.3%)	2 (33.3%)		0 (0.0%)	0 (0.0%)		0 (0.0%)	1 (11.1%)	
Unsmooth	3 (100.0%)	9 (60.0%)		29 (96.7%)	4 (66.7%)		20 (100.0%)	3 (100.0%)		18 (100.0%)	8 (88.9%)	
Posterior acoustic pattern			0.167			0.643			0.253			1.000
No-shadowing	2 (66.7%)	15 (100.0%)		20 (66.7%)	5 (83.3%)		11 (55.0%)	3 (100.0%)		13 (72.2%)	6 (66.7%)	
Shadowing	1 (33.3%)	0 (0.0%)		10 (33.3%)	1 (16.7%)		9 (45.0%)	0 (0.0%)		5 (27.8%)	3 (33.3%)	
Calcification			1.000			0.109			1.000			1.000
Absent	1 (33.3%)	7 (46.7%)		5 (16.7%)	3 (50.0%)		8 (40.0%)	1 (33.3%)		4 (22.2%)	2 (22.2%)	
Present	2 (66.7%)	8 (53.3%)		25 (83.3%)	3 (50.0%)		12 (60.0%)	2 (66.7%)		14 (77.8%)	7 (77.8%)	
Echo pattern			0.314			0.024*			1.000			0.538
Hypoechoic	1 (33.3%)	1 (6.7%)		2 (6.7%)	3 (50.0%)		1 (5.0%)	0 (0.0%)		2 (11.1%)	0 (0.0%)	
Heterogeneous	2 (66.7%)	14 (93.3%)		28 (93.3%)	3 (50.0%)		19 (95.0%)	3 (100.0%)		16 (88.9%)	9 (100.0%)	
Adler grade			1.000			1.000			0.229			1.000
0–2	1 (33.3%)	6 (40.0%)		15 (50.0%)	3 (50.0%)		10 (50.0%)	3 (100.0%)		1 (5.6%)	0 (0.0%)	
3	2 (66.7%)	9 (60.0%)		15 (50.0%)	3 (50.0%)		10 (50.0%)	0 (0.0%)		17 (94.4%)	9 (100.0%)	
Elasticity score			1.000			0.374			0.155			1.000
2–3	2 (66.7%)	7 (46.7%)		12 (40.0%)	4 (66.7%)		4 (20.0%)	2 (66.7%)		4 (22.2%)	2 (22.2%)	
4–5	1 (33.3%)	8 (53.3%)		18 (60.0%)	2 (33.3%)		16 (80.0%)	1 (33.3%)		14 (77.8%)	7 (77.8%)	
BI-RADS			1.000			0.643			1.000			0.683
3-4a	1 (33.3%)	3 (20.0%)		10 (33.3%)	1 (16.7%)		5 (25.0%)	1 (33.3%)		9 (50.0%)	3 (33.3%)	
4b-5	2 (66.7%)	12 (80.0%)		20 (66.7%)	5 (83.3%)		15 (75.0%)	2 (66.7%)		9 (50.0%)	6 (66.7%)	

*TNBC*, triple-negative breast cancer; *HER2*, human epidermal growth factor receptor 2; *AR*, androgen receptor; *BI-RADS*, breast imaging reporting and data system; * indicates *p* < 0.05

### The nomogram for predicting AR status

#### Construction of logistic regression model

**Fig. 5:**
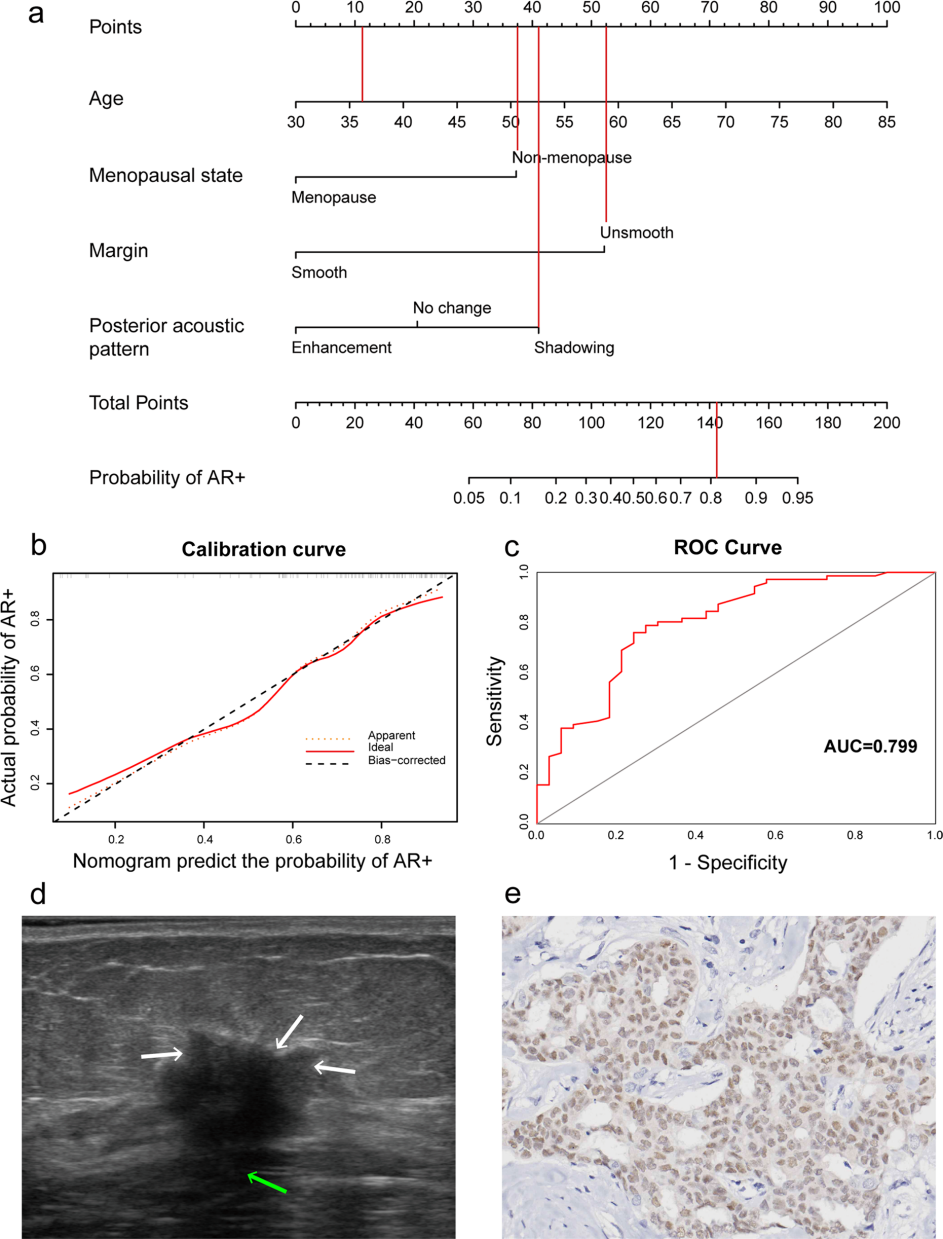
An application example of nomogram to predict the AR status. **a** A nomogram was constructed from four informative features. **b** Calibration curve for evaluating nomogram effectiveness fitted well. **c** Receiver operating characteristic curve of the model for predicting AR status. **a** and **d** In a 36-year-old (11 points) non-menopausal (37.5 points) woman with breast cancer, the B-mode ultrasound showed the tumor was 15 mm in diameter, the margin of the tumor was unsmooth (52.5 points), and the posterior acoustic pattern was shadowing (41 points) and BI-RADS 4c. The total points were about 142 and the probability of AR + predicted by our nomogram was about 82% in this case. **e** The immunohistochemistry result of the patient: nuclear staining was more than 10%, the expression of AR was positive, original magnification × 400. *ROC*, receiver operating characteristic; *AUC*, area under curve; *AR*, androgen receptor

We included the clinical features available before surgery (age, tumor size and menopausal status) and statistically significant ultrasound features (margin, posterior acoustic pattern and elasticity score) in the multivariate logistic regression analysis (Table 4). The results showed that age (OR: 1.105 (1.023, 1.194), *p* = 0.011), posterior acoustic pattern (OR: 2.930 (1.193, 7.192), *p* = 0.019) and margin (OR: 14.984 (1.625, 138.161), *p* = 0.017) were positively correlated with the expression of AR, while menopausal status (OR: 0.135 (0.029, 0.634), *p* = 0.011) was negatively correlated. This meant that the probability of AR positive expression was higher in patients who were older, with posterior acoustic shadowing, with unsmooth margins, and non-menopause. Based on the statistically significant variables of multivariate regression, the final logistic model was established to predict the AR status. The model showed excellent diagnostic efficiency with the Hosmer-Lemeshow goodness of fit test for AR (*χ*^2^ = 3.545, *p* = 0.896), and AUC of 0.799 (Fig. 5c).

**Table 4:** Multivariate logistic regression analysis of selected variables (clinical features and ultrasound characteristics)

Variables	*β*	S.E	Wald	*p* value	Odds radio	95% LCL	95% UCL
Menopausal status	− 1.999	0.787	6.451	0.011*	0.135	0.029	0.634
Tumor size	− 0.019	0.025	0.557	0.455	0.981	0.934	1.031
Age	0.100	0.039	6.426	0.011*	1.105	1.023	1.194
Elasticity score	− 0.013	0.38	0.001	0.974	0.987	0.469	2.081
Posterior acoustic pattern	1.075	0.458	5.503	0.019*	2.930	1.193	7.192
Margin	2.707	1.133	5.704	0.017*	14.984	1.625	138.161

*LCL*, lower control limit; *UCL*, upper control limit; * indicates *p* < 0.05

#### Development and validation of nomogram

Subsequently, the nomogram was constructed according to the logistic regression model. In the nomogram, the menopausal status, age, margin and posterior acoustic pattern were distributed according to their risk coefficient. Each patient can calculate the total points by accumulating the corresponding points of each feature according to their conditions to evaluate the AR status (Fig. 5a). There was no obvious deviation in the calibration curve, with the C-index of 0.799, which showed the nomogram performed well (Fig. 5b). An example of the nomogram application is shown in Fig. 5. The patient's total points were approximately 142, and the probability of AR + was 82%. The result of IHC confirmed that the expression of AR was positive.

### The correlation between the expression of AR and PFS in breast cancer

**Fig. 6:**
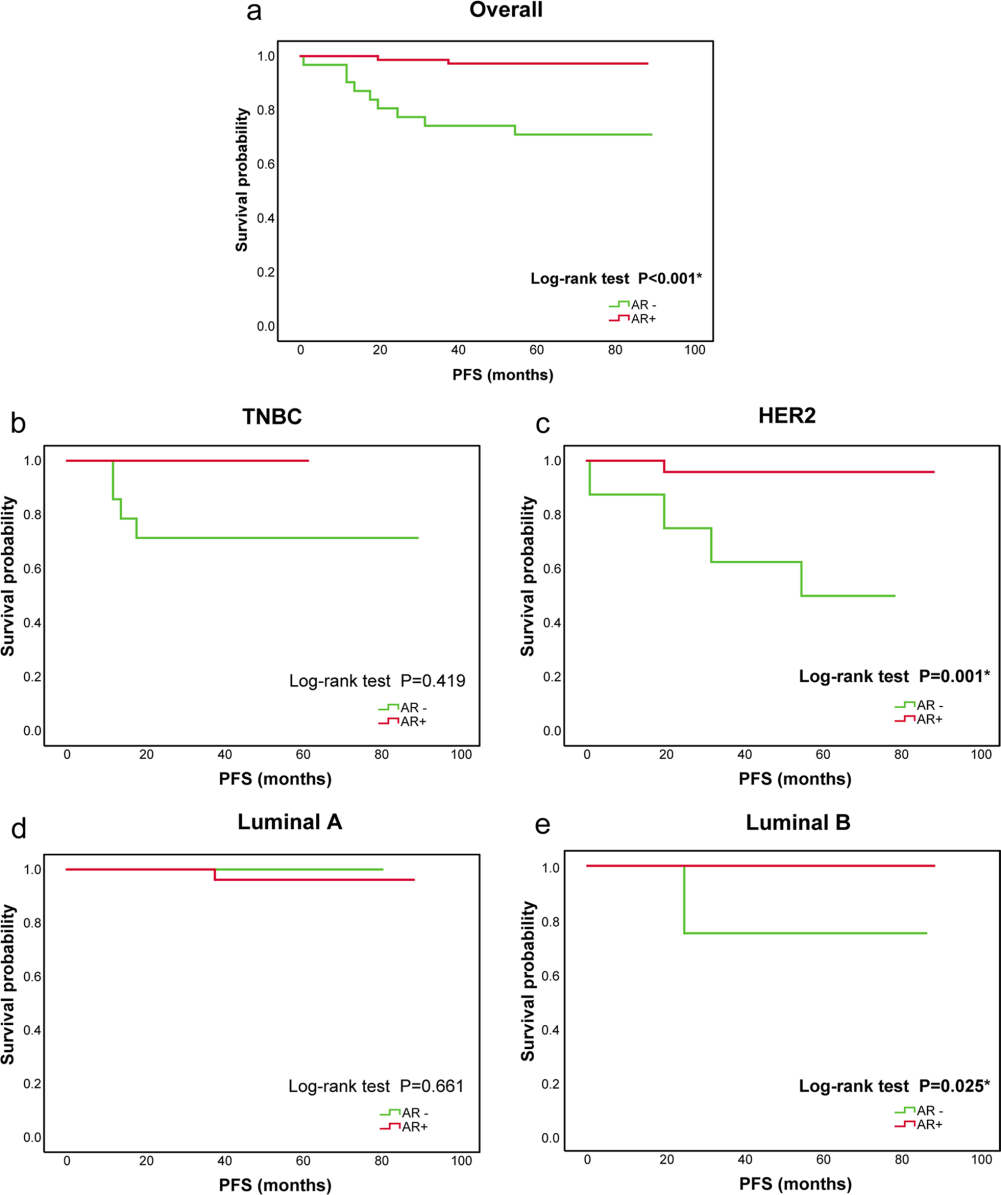
Prognostic role of AR in different breast cancer patients. **a** Overall patients (*n* = 103), 2 cases (2.78%,2/72) in the AR + group vs 9 cases (29.03%,9/31) in the AR- group experienced disease progression. **b** TNBC patients(*n* = 16), no patient (0/2) in the AR + group versus 4 cases (28.57%,4/14) in the AR– group experienced disease progression. **c** HER2 overexpression subtype patients(*n* = 32), 1 case (4.17%,1/24) in the AR + group versus 4 cases (50%,4/8) in the AR- group experienced disease progression. **d** Luminal A subtype patients(*n* = 31), 1 case (3.85%,1/26) in the AR + group versus no patient (0/5) in the AR– group experienced disease progression. **e** Luminal B subtype patients(*n* = 24), no patient (0/20) in the AR + group versus 1 case (25%,1/4) in the AR– group experienced disease progression. *TNBC*, triple negative breast cancer; *HER2*, human epidermal growth factor receptor 2; *AR*, androgen receptor; *PFS*, progression-free survival; * indicates *p* < 0.05

In this study, a total of 103 patients (AR +  = 72, AR– = 31) were followed up from 1 to 90 months (median, 63 months). Of the 103 patients, 11 cases experienced disease progression. Only 2 patients (2.78%, 2/72) in the AR + group experienced disease recurrence, compared with 9 (29.03%, 9/31) in the AR– group. The 5-year survival rate of AR + and AR– groups was 97.22% and 70.97% for PFS, respectively. After the Log-rank test, there was a statistically significant difference between the two groups (χ^2^ = 16.895, *p* < 0.001). According to AR status, the Kaplan–Meier plot of PFS for the entire study cohort is presented in Fig. 6a. In general, it can be observed that the prognosis of patients with AR + was significantly better than that of AR– (Additional file 1: Fig. S1). In addition, the results showed that AR status (HR: 0.135 (0.028, 0.664), *p* = 0.014) and tumor size (HR: 1.103 (1.048, 1.162), *p* < 0.001) were independent prognostic factors for breast cancer by multivariate Cox regression analysis. In the survival analysis of different subtypes, it was found that the clinical outcomes of Luminal B (*p* = 0.025) and HER2 overexpression subtypes (*p* = 0.001) were consistent with that of the entire study cohort; the patients with AR + had favorably clinical outcomes (Fig. 6b– e).

## Discussion

It is generally acknowledged that AR plays an important role in the physiology and pathology of men; nevertheless, it also counts a great deal in female breast cancer. Many studies have proven that gene expression can affect the imaging performance of tumors.[13, 30] Previous studies on AR have been done on the imaging of TNBC [16–18]. However, all molecular subtypes were included in our study, and there were significant differences in clinicopathological features and ultrasound characteristics between AR– and AR + groups in the entire study cohort. Then, there were also variations between the AR– and AR + breast cancer in different subtypes, grouped by molecular subtype. Finally, we also found that AR + breast cancer patients tend to have better prognosis.

Consequently, AR + breast cancer usually had lower histological grade and lower Ki-67 level. The expression of AR was also related to the molecular subtypes of breast cancer. Ki-67 exists in proliferating cells and can evaluate breast cancer prognosis as a marker of proliferation [31]. The higher the Ki-67 index, the faster the tumor proliferation and the worse prognosis of patients [32]. The histological grade is also an important factor in evaluating the prognosis. Tumors with high histological grade usually proliferate rapidly and differentiate poorly, indicating adverse clinical outcomes [33]. Consequently, it can reveal that the tumor with AR + can obtain better clinical results by inhibiting cell proliferation, which is consistent with the results of our survival analysis. We also found that the probability of AR + in TNBC was the lowest, just accounting for 18.18%, compared with HER2 overexpression (73.81%), Luminal A (85.71%) and Luminal B (88.57%). It may be related to the high degree of malignancy, strong invasiveness, rapid development, easy recurrence and low survival rate of TNBC.

As we can see in the research, AR + tumors usually show unsmooth margin, posterior acoustic shadowing and higher elasticity score (that is, stiffer) on ultrasound. Ultrasound elastography can semi-quantitatively measure the hardness of tumor in a routine examination [34]. The hardness of tumors is a characteristic of the extracellular matrix regulated by collagen cross-linking [35]. It has been found that there is insufficient vessel supply in stiffer tumors, which leads to the proportion of cancer cells decreases and is replaced by fibrosis. Therefore, the tumor showed a higher elasticity score on ultrasound [36]. Our results showed that AR + breast tumors were stiffer, which indicates that a higher degree of fibrosis and a relatively lower proportion of cancer cells in the AR + group lead to the slow progression of cancer. The posterior acoustic pattern is determined by the proportion of fibrous tissue and breast glands in the tumor. Posterior acoustic shadowing is caused by the decrease in sound beam penetration due to the proliferation of connective tissue, which is usually regarded as a sign of lower-grade malignant tumors [37]. The ultrasound findings of the margins of masses with good prognosis are controversial. Some researchers suggested that unsmooth margin is highly related to a malignant tumor with a poor prognosis [38]. However, we found that most AR + tumors showed unsmooth margins and better prognosis, which was supported by many studies [39]. Some studies have argued that the proliferation in varying degrees of collagen fibers around breast tumors leads to unsmooth margin on ultrasound images, which may inhibit the rapid infiltration of tumor cells [35]. Hence, the lower-grade malignant tumors tend to show unsmooth margin, the higher-grade tumors may show smooth margin [39, 40]. Therefore, it is clear that AR can be seen as a marker of a favorable outcome in breast cancer. Perhaps because the sample size was not large enough, we did not find more significant differences in ultrasound performance between the AR– and AR + groups in each molecular subtype; notwithstanding, only one distinctive characteristic was found in the Luminal A subtype. The echo pattern of tumor is related to fibrosis, cellular components and necrosis [41]. Some studies have found that the internal echo of locally advanced tumors showed hypoechoic [42]. Our results showed that AR– group was more likely to show homogeneous hypoechoic in Luminal A breast cancer, which indicates that the malignant degree of AR– group is higher than AR + group. Taken together, further investigation of this finding is warranted.

We initially constructed a simple nomogram to predict the AR status based on our findings that AR is indeed related to ultrasound and clinicopathological features. Nomogram is a model that can reflect the evaluation value intuitively, and contains various characteristics. It can not only be used to the prediction, but also to verify the correlation [43]. Our nomogram was established according to menopausal status, margin, posterior acoustic pattern and age, easily obtained in preoperative diagnosis and treatment. The nomogram demonstrated satisfactory performance with a C-index of 0.799, which proved the close correlations between the selected features and AR status. This is the main purpose to construct the nomogram in our study. Meanwhile, the initially successful construction of the nomogram also plays a solid foundation for the further study. With the in-depth study of AR in breast cancer, AR–related therapy is gradually applied in clinical practice [44]. In future studies with larger samples, we will focus on non-invasive and rapid prediction of AR status by clinical and imaging characteristics before surgery. This will provide valuable information for the formulation of clinical treatment plans.

Survival analysis showed that AR status and tumor size were independent prognostic factors of PFS. Besides, in the entire study cohort, the clinical outcomes of AR + patients were significantly better than that of AR–. Moreover, we analyzed the effect of the AR status on prognosis in different subtypes and found that the results in HER2 overexpression and Luminal B subtypes were consistent with the overall results, which were in accordance with the results of Jiang et al.[45]. Given experimental data have shown cross-talk between AR and HER2 pathways, which can influence the prognosis of HER2 + breast cancer [44]. However, our study showed that there was no significant difference in prognosis between the AR– and AR + groups in the TNBC and Luminal A subtypes. In our study, the cohort of TNBC was the smallest, with only 16 cases, and the AR positive expression rate of TNBC was also the lowest among the four molecular subtypes. Meanwhile, Gao et al. [46] suggested that Luminal A subtype had the best prognosis among the four molecular subtypes. Our research also found that only one of 31 Luminal A patients had recurrent disease, which supports the above view. Therefore, this study did not find the effect of AR status on the prognosis of TNBC and Luminal A subtypes. However, experiments have found that AR can interfere with ER binding to estrogen-related elements and inhibit the proliferative effect of ER, thereby promoting cancer cell apoptosis [47]. Another study has also shown that the patients with AR + TNBC had better survival outcomes [48]. Taken together, these studies indicate that AR is important in each molecular subtype of breast cancer, and therefore, further exploration is urgently needed.

Altogether, we studied the correlation between AR status and ultrasound, clinicopathological features and clinical outcomes in breast cancer, although our research still has some limitations. First, the sample size is not large enough, which might lead to bias in the results. Our study can be regarded as the initial exploration, and the sample size should continue to be expanded to analyze different results caused by the expression of AR in different subtypes. In order to explore whether AR is related to more detailed categorization of BI-RADS, it is also necessary to expand the sample size. Second, we did not include more features, such as the number of lymph node metastasis, peritumoral vascular invasion and blood flow resistance index, among others. And we only included strain elastography, without evaluating shear wave elastography. Additionally, mammogram, MRI and other imaging features were not included in this study. Then, we did not evaluate the diagnostic differences between radiologists. Finally, the simple nomogram was mainly used to verify the correlation, and the subsequent research will improve it for better prediction.

In conclusion, AR is closely related to the clinicopathological features and prognosis of breast cancer. Moreover, the ultrasound findings of breast cancer with different expression of AR are also different. As a new molecular marker of breast cancer and an important prognostic factor, AR will play an increasingly important role in diagnosing and treating breast cancer. The above results will help to better understand various biological functions of AR and provide more information for the treatment of breast cancer.

## Supplementary Information

**Additional file 1. Fig. S1**. Examples of ultrasound and IHC images of two patients with breast cancer.

## Data Availability

The imaging data and code that support the findings of this study are available from the corresponding author upon request.

## Abbreviations

AR: Androgen receptor
ASCO/CAP: American Society of Clinical Oncology/College of American Pathologists
AUC: Area under curve
BI-RADS: Breast Imaging Reporting and Data System
CI: Confidence interval
C-index: Concordance index
ER: Estrogen receptor
FISH: Fluorescence in situ hybridization
HER2: Human epidermal growth factor receptor 2
HR: Hazard ratio
IHC: Immunohistochemistry
LAR: Luminal androgen receptor
OR: Odds ratio
PFS: Progression-free survival
PR: Progesterone receptor
ROC: Receiver operating characteristic curve
TMA: Tissue microarray
TNBC: Triple-negative breast cancer
WFUMB: World Federation of Ultrasound in Medicine and Biology
