# Supplementary material for: Correlation of androgen receptor with ultrasound, clinicopathological features and clinical outcomes in breast cancer
Source: Insights Imaging. 2023 Mar 16;14:46. doi: 10.1186/s13244-023-01387-9 (PMC10020396; doi:10.1186/s13244-023-01387-9)
Supplement: Supplementary file 1 — Additional file 1. Fig. S1. Examples of ultrasound and IHC images of two patients with breast cancer. [file 13244_2023_1387_MOESM1_ESM.pdf]

ELECTRONIC SUPPLEMENTARY MATERIAL

Correlation of androgen receptor with ultrasound, clinicopathological features and clinical outcomes in breast cancer

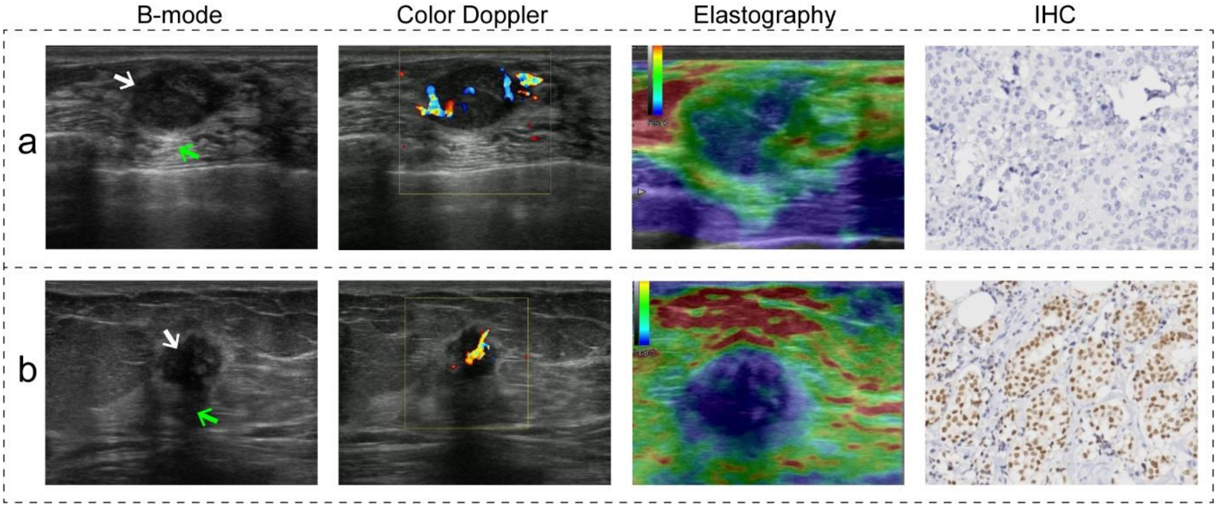

Fig. S1: Examples of ultrasound and IHC images of two patients with breast cancer. (**a**) B-mode, Color Doppler, Elastography and IHC images of a woman with triple negative breast cancer in the left breast, 58 years old, who had experienced recurrence at the 12th month after operation. B-mode: associated with posterior acoustic enhancement (green arrow) and smooth margin (white arrow). Color Doppler: showed rich color flow signal on the circumference and inside tumor (Adler grade was 3). Elastography: with the center was blue and the periphery was green, the tumor was relatively hard (the elastography score was 3). IHC staining of AR: nuclear staining <10%, the expression of AR was negative, original magnification × 400. (**b**) B-mode, Color Doppler, Elastography and IHC images of a woman with luminal A subtype breast cancer in the right breast, 62 years old, who had not yet experienced disease recurrence. B-mode: associated with posterior acoustic shadowing (green arrow) and unsmooth margin (white arrow). Color Doppler: showed rich color flow signal (Adler grade was 3). Elastography: with shaded blue, the tumor was hard (the

elastography score was 4). IHC staining of AR: nuclear staining >10%, the expression of AR was positive, original magnification ×400.

IHC, immunohistochemistry; *AR*, androgen receptor.
